# Effects of 10-Year Management Regimes on the Soil Seed Bank in Saline-Alkaline Grassland

**DOI:** 10.1371/journal.pone.0122319

**Published:** 2015-04-22

**Authors:** Hongyuan Ma, Haoyu Yang, Zhengwei Liang, Mark K. J. Ooi

**Affiliations:** 1 Northeast Institute of Geography and Agroecology, Chinese Academy of Sciences, Changchun, China; 2 Da’anSodic Land Experiment Station of China, Da’an, China; 3 Institute for Conservation Biology, School of Biological Sciences, University of Wollongong, Wollongong, Australia; University of New South Wales, AUSTRALIA

## Abstract

**Background:**

Management regimes for vegetation restoration of degraded grasslands can significantly affect the process of ecological succession. However, few studies have focused on variation in the soil seed bank during vegetation restoration under different management regimes, especially in saline-alkaline grassland habitats. Our aim was to provide insights into the ecological effects of grassland management regimes on soil seed bank composition and vegetation establishment in mown, fenced, transplanted and natural grassland sites, all dominated by the perennial rhizomatous grass *Leymus chinensis*.

**Methodology:**

We studied species composition and diversity in both the soil seed bank and aboveground vegetation in differently managed grasslands in Northeast China. An NMDS (nonmetric multidimensional scaling) was used to evaluate the relationship between species composition, soil seed banks, aboveground vegetation and soil properties.

**Principal Findings:**

Fenced and mown grassland sites had high density and species richness in both the soil seed bank and aboveground vegetation. The Transplanted treatment exhibited the highest vegetation growth and seed production of the target species *L*. *chinensis*. Seeds of *L*. *chinensis* in the soil occurred only in transplanted and natural grassland. Based on the NMDS analysis, the number of species in both the soil seed bank and aboveground vegetation were significantly related to soil Na^+^, Cl^-^, RSC (residual sodium carbonate), alkalinity, ESP (exchangeable sodium percentage) and AP (available phosphorus).

**Conclusions:**

Soil seed bank composition and diversity in the saline-alkaline grassland were significantly affected by the management regimes implemented, and were also significantly related to the aboveground vegetation and several soil properties. Based on vegetative growth, reproductive output and maintenance of soil seed bank, the transplanting was identified as the most effective method for relatively rapid restoration of the target species *L*. *chinensis*. This approach could be beneficial for the restoration of dominant species in a wide range of degraded grassland ecosystems.

## Introduction

As a consequence of global environmental changes and human utilization, many of the world’s ecosystems have degraded since the middle of the 20th century, including large proportions of grasslands [1–4], wetlands and coastal saline marshes [5, 6] and forests [7]. Restoration of these degraded ecosystems is an increasingly relevant focus of current ecological research [8,9]. Various management regimes are carried out to facilitate restoration, including mowing instead of grazing [10], fencing [11,12], reseeding or transfer of seed-containing plant material [8,13], or transplanting of rhizomatous tillers or seedlings of plants [14,15]. It is especially crucial to investigate the variation in response of both ecosystem structure and biodiversity under different management treatments, to subsequently assess and inform future restoration actions, plans and implementation. However, although some studies have investigated the impact of management on above-ground vegetation, few studies have examined the effects on their corresponding seed bank characteristics.

Soil seed banks represent a stock of regeneration potential in many plant communities as well as represent an important component of ecosystem resilience [16]. Although clonal spread may play an important role in some stressful environments [17], the soil seed bank can play a crucial role in vegetation dynamics in degraded ecosystems. In particular, soil seed banks allow species to bridge temporally unsuitable habitat conditions for germination and establishment [17]. Insight into the mechanisms maintaining natural community dynamics may result from understanding of soil seed bank and soil or vegetation relationships, which in turn will contribute to improving land management practices [13,18].

Previous studies have indicated that different management practices are an important factor affecting soil seed bank composition, although their conclusions have varied substantially. Shang et al. [10] found large differences in seed bank density and composition between different management treatments of grazing, mowing and abandonment for 11 years, in which the grazed plots showed the highest densities and the abandoned plots the lowest. However, Milberg [19] and Bakker et al. [20] found no significant differences in seed bank density and species richness between grasslands and spontaneously established forests or shrublands 18 years after grazing abandonment. Koch [21] found that seed bank development lagged behind that of the associated vegetation, but that the relationship reached “equilibrium” after several decades. While the above references are examples of studies that have investigated the effects of different management regimes carried out in grassland restoration, limited information is available on the relative response of vegetation to multiple management approaches such as mowing, fencing and transplantation, or on how this response relates to seed bank composition.

Management changes can affect plant reproduction and soil physio-chemical characteristics, which will further affect the soil seed bank composition. Brys et al. [22] found that abandonment led to a strong decline in species richness and reduced flowering performance of the remaining plants. In addition, livestock grazing on plant reproductive parts [23], heavy grazing intensity [24] and high mowing frequency [25] might contribute to explain reduced seed set and production. Seed distribution patterns and seed persistence in the soil are affected by soil properties such as particle sizes, structure and soil chemistry [26,27]. Recruitment from the seed bank is restricted to periods with favorable soil conditions, through controlling seed germination [28]. However, the relationship between soil seed bank and vegetation and/or soil characteristics is not fully understood, especially in sensitive habitats with seriously degraded soil conditions, such as saline-alkaline grasslands [12].

Saline-alkaline grasslands are always characterized by high pH (9.55–9.87 in [27]), Na^+^, ESP (exchangeable sodium percentage), alkalinity, and low EC (electrical conductivity, 0.13–0.33 mS/cm in this study, 0.98–2.8 mS/cm in [30]; 0.1–3.2 mS/cm in [31]), SOM (soil organic matter), and AN (available nitrogen) [29]. Soil alkalinity and salinity is thought to be one of the major factors determining seed germination and dormancy [32], as well as the aboveground vegetation composition [31], seed production and seed dispersal [13]. Most previous studies of the factors which control soil seed bank characteristics have focused on coastal and inland salt wetlands, such as marsh, meadow and wet grassland [33,34]. However, much less is known about soil seed bank variation in inland saline-alkaline grassland [30,35].

The Songnen grassland, part of the Euro-Asia steppe dominated by the perennial grass *Leymus chinensis* (Trin.) Tzvelez, is one of the most important grazing and mowing pastures in China. However, due to human interference, especially overgrazing, the *L*. *chinensis* grassland has degraded seriously to unprecedented levels. There are approximately 3.73×10^6^ ha of saline-alkaline land in Northeastern China and the area of saline-alkalinity has increased at 0.6% per year in the past 50 years [29]. Most of the original *L*. *chinensis* grassland in this region is degraded to varying degrees [32] and its structural and functional loss has become an acute problem for ecological restoration. It is therefore urgent that the restoration and rehabilitation of this grassland is the focus of research, especially with the target of restoring the key species *L*. *chinensis*. Recently, management methods, such as mowing instead of grazing, fencing, transplanting, and abandonment have resulted in successful revegetation of *L*. *chinensis* in severely degraded grasslands in the Songnen plain [36,37]. However, in contrast to the development of aboveground vegetation, there is a paucity of information on the current state of the soil seed bank of these restored grasslands under different management regimes.

In order to assess the restoration potential of degraded *L*. *chinensis* grasslands, this study examined the density and species richness of both the soil seed bank and vegetation in four management types that had been implemented for approximately 10 years. These included a continuously mown treatment (Mown), a fenced treatment (Fenced), a transplanted grassland (Transplanted) and a natural grassland without mowing or grazing (Natural, as control). We also determined the soil properties and the growth of vegetation. We addressed the following questions: (i) What is the response of the soil seed bank to the management regimes applied? (ii) Do the management regimes affect the growth and seed production of the dominant species *L*. *chinensis*? (iii) Which parameters of the saline-alkaline soil influence the composition of the soil seed bank and aboveground vegetation under different management regimes? (iv) In relation to the soil seed bank, which management regime is better for the restoration *L*. *chinensis*? The results obtained in this study will provide insights into the ecological influence of management regimes on vegetation establishment within degraded ecosystems.

## Materials and Methods

### Ethics statement

No permits were required to carry out this study. The owner of all the study sites is the P.R China. The Chinese government has given us permission to conduct the study on these sites. We confirm that the field studies did not involve endangered or protected species and that no vertebrate studies were studied in this project.

### Study areas and sites

The study was conducted in the degraded grassland of the Songnen Plain, northeastern China (N42°30′-51°20′, E121°40′-128°30′). This region straddles the semi-humid and semi-arid continental monsoon climatic boundary. The annual average temperature is 4.7°C, with daytime averages ranging from -17.6°C in January, to 23.6°C in July [38]. The annual average precipitation is 410 mm, 70–80% of which occurs from July to September. The average evaporation in this region is 1790 mm, four times greater than the precipitation. During the study period, data recorded by the Da’an Sodic Land Experiment Station showed that rainfall and evaporation were 185.7 mm and 1525.0 mm respectively in 2010, and 374.0 mm and 1511.7 mm respectively in 2011. Seasonal drought is frequent in spring and autumn, with drought occurring 90% of the time in spring. These conditions have resulted in salts accumulating throughout the soil profile. The sources of salts in this region include abundant Na_2_CO_3_ and NaHCO_3_ from the weathering and a series of chemical reactions of the albite and aluminosilicate in the magmatic rocks [38]. The soil pH is 8.5–11.0. Species distribution patterns and plant growth depend on the degree of salinity-alkalinity in the soil. *Leymus chinensis* is the dominant species in the area where the soil is lightly degraded, accompanied by some annual or biannual species e.g. *Chloris virgata* Sw., *Puccinellia chinampoensis* Ohwi., *Suaeda salsa* Pall., *Kochia sieversiana* (Pall.) C.A. Mey., *Artemisia anethifolia* Weber ex Stechm. and *Polygonum sibiricum* Laxm.

Our investigations were carried out in four habitats following application of different management regimes, i.e. Mown, Fenced, Transplanted and Natural treatments for 10 years, beginning in 2002. Before restoration, cattle and sheep grazed these grasslands for decades with the exception of the natural treatment which had remained relatively undisturbed. The mown habitat (2.5×10^4^ ha), was mown once a year in August using agricultural machinery, with the hay subsequently removed. In the Fenced habitat (0.5×10^4^ ha), the grassland had been fenced with wire netting approximately 1 m high to exclude the main grazing animals such as sheep and cattle and encourage natural restoration without any mowing or grazing. The Transplanted habitat (1.0 ha) was established by transplanting adult plants of *L*. *chinensis* in 2002 and natural succession was followed afterwards. For the transplant treatment, we used hand planting. Small holes about 20 cm apart were made in ploughed rows which were spaced 40 cm apart. Three tillers *of Leymus chinensis* with rhizomes were planted in each hole. After transplanting, the land was irrigated with a one-off watering treatment. *Leymus chinensis* growth was visible within 2–3 weeks of watering. All treatments were applied to sites within the Songnen Plain, with the Transplanted and natural treatments distributed within a 100 ha site at the Da’an Sodic Land Experimant Station of China, and the Fenced and Mown treatments distributed outside of this station.

### Soil samples

Soil samples of the seed bank were collected in April 2011, before seed germination in the field. In each of the four habitats, three randomly selected sites (6 m × 6 m) were established. To smooth out the core-to core variation within each site, 12 soil cores were extracted from 0–5 cm depth (based on our preliminary experiment which showed that the soil seed mainly concentrated on the top layer of 0–5 cm) using a soil corer (2.5 cm diameter) and then mixed together to form a single sample per site. The samples were carried in plastic bags to the laboratory and processed using the methodology as described by Ter Heerdt et al. [39]. Large soil aggregates were broken up and soil samples were homogenized. Each soil sample was washed through a coarse (4 mm) and a fine (0.2 mm) sieve in order to remove rhizomatous parts, root fragments, and coarse soil material.

### Seedling germination assay

The aggregated soil samples were spread out evenly in 1 cm thick layers on 20 cm × 15 cm × 8 cm plastic trays filled with 6 cm depth sterile vermiculite (sterilized at 120°C for 24h). The trays were placed on shelves in a greenhouse. The temperature and lighting were not controlled, naturally varying between 15°C and 28°C and 12 h to 14 h respectively. Ten control trays filled only with sterilized vermiculite were also placed randomly on the shelves to test for contamination. No seedlings germinated in the control trays. Soil samples were watered daily to ensure the soil remained moist, yet not water-logged. Germinated seedlings were identified based on Fu et al. [40] and removed as soon as possible after germination. Seedlings that could not be identified immediately were replanted into pots and grown until they could be identified. When germination ceased, the soil samples were broken up to promote the germination of any remaining seeds. This was conducted every four weeks for five months, after which the experiment was terminated because no new seedlings emerged.

### Vegetation survey and seed production of *Leymus chinensis*

A vegetation survey was performed in July 2011 during the peak of the growing season in the four treatment sites. Three 1m^2^ quadrats were randomly placed in each site. Species presence and abundance (number of individuals) within each quadrat were recorded. The height of the target species *L*. *chinensis* in each habitat was measured in July. To measure reproductive output, we randomly selected 30 spikelets of *L*. *chinensis* from each site of each habitat and counted the number of seeds produced on each spikelet. However, in the Mown habitat, no spikelets of *L*. *chinensis* were observed.

### Soil properties analysis

During the period in which aboveground vegetation was surveyed, three soil samples of 5 cm diameter × 10 cm depth were collected from each site where the soil seed bank was sampled. All soil samples were air-dried and then passed through a 2 mm sieve. Soil available nitrogen (AN) and available phosphorus (AP) was determined after extraction with potassium chloride, and sodium bicarbonate, respectively. Soil organic carbon (SOC) was determined by the Total Organic Carbon Analyzer Model TOC-VCPH (Shimadzu, Japan). Soil pH and EC (soil/water = 1:5, w/v) were measured by a digital PHS-3C pH meter and a DDS-307 conductivity meter respectively. Soluble salt estimates were based on 1:5 soil-water extracts. The Na^+^, K^+^, Ca^2+^, and Mg^2+^ were determined using induced couple-plasma spectroscopy (GBC, Scientific Equipment Pty Ltd., Australia) and Cl^-^, NO_3_^-^, HCO_3_^-^, CO_3_^2-^ were determined by standard methods [29, 41]. Alkalinity and residual sodium carbonate (RSC) were calculated as follows: alkalinity = (HCO_3_^-^+CO_3_^2-^), RSC = (HCO_3_^-^+CO_3_^2-^)-(Ca^2+^+Mg^2+^). Sodium adsorption ratio (SAR) and ESP were calculated by the equations in [41].

### Statistic analysis

A one-way ANOVA and a subsequent Tukey’s test were used to test for differences between the four management regimes for the factors species richness and seed density of the soil seed bank, species diversity of the soil seed bank and aboveground vegetation (based on the Shannon-Weaver index as described in [41]), plant height, the amount of seed set, and also for all soil variables. Normality of the data and homogeneity of variances were checked and, if necessary, the data were appropriately transformed to satisfy the assumptions of ANOVA. Results were considered statistically significant at α = 0.05. All statistical analyses were conducted with the software SPSS 21.0 for Windows (SPSS Inc., Chicago, USA).

Similarity of species composition between the seed bank and the vegetation within each habitat was tested by non-metric multidimensional scaling (NMDS) with a Bray-Curtis distance measure using the metaMDS function in the Vegan package in R version 3.0.2 [42]. The resulting NMDS axis values are produced such that sites with similar species composition lie close to one another in ordination space. To assess the relationships of density within the soil seed bank or species abundance of aboveground vegetation with environmental variables, multivariate analysis was performed with a two-dimensional NMDS using a Euclidian dissimilarity matrix. Environmental vectors were examined based on continuous variables including SOC, AN, AP, TK, Na^+^, Cl^-^, SO_4_^2-^, pH, EC, RSC, Alkalinity, SAR and ESP. Data were centered and scaled prior to the analysis [5], which was carried out using the Vegan Package in R [43].

## Results

### Soil seed bank composition

**Fig 1:**
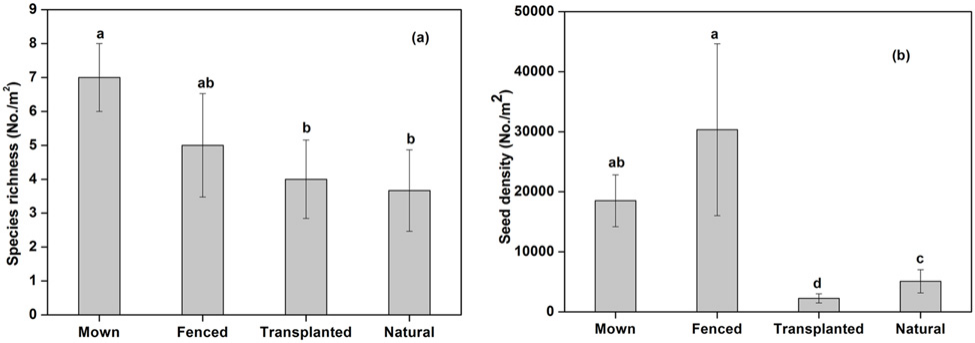
Soil seed bank composition in the Songnen grassland, northeastern China, including (a) species richness and (b) seed density. Management regimes included Mown, Fenced, Transplanted and Natural (as control). Bars with the same lower case letters indicate no significant differences (p > 0.05) between different management regimes based on Tukey’s test. Error bars are mean ± s.e.

A total of 16 species germinated from the soil (Table 1), which belonged to seven families (Poaceae, Asteraceae, Fabaceae, Chenopodiaceae, Rosaceae, Cyperaceae, and Violaceae) with the dominant family being Poaceae. There were nine perennial species, two biennial and five annual herbs. The mean species richness per habitat showed no significant differences among the four habitats (Fig 1a, F_3,8_ = 1.199, p > 0.05, Table 2). The seed density within the soil seed banks were significantly different under different management regimes (Fig 1b, F_3,8_ = 12.794, p < 0.01, Table 2). The highest mean seed density of 30348/m^2^ appeared in the Fenced treatment while the lowest of 2265 seeds/m^2^ was found in transplanted grassland. The seeds of climax species *L*. *chinensis* appeared in the transplanted and natural grasslands with a density of 679 seeds/m^2^ and 736 seeds/m^2^ respectively.

**Table 1:** Seed density and species richness of the soil seed bank under each management treatment of Mown, Fenced, Transplanted and Natural grasslands in the Songnen grassland, Northeastern China, in April 2011 (No./m^2^).

Species	Mown	Fenced	Transplanted	Natural
*Leymus chinensis*	0±0	0±0	736±150	679±259
*Puccinellia chinampoensis*	0±0	16249±9814	57±57	0±0
*Chloris virgata*	3454±247	10361±4817	170±170	226±226
*Setaria viridis*	2378±643	1132±1132	0±0	0±0
*Chenopodium stenophyllum*	226±226	0±0	0±0	1076±906
*Suaeda salsa*	623±623	0±0	0±0	0±0
*Sonchus arvensis*	0±0	0±0	113±113	453±453
*Scirpus triqueter*	9172±3254	0±0	0±0	0±0
*Sonchus oleraceus*	453±371	57±57	962±247	0±0
*Viola dissecta*	0±0	0±0	0±0	2345±1594
*Potentilla flagellaris*	906±743	793±709	57±57	0±0
*Gueldenstaedtia verna*	113±57	0±0	0±0	226±226
*Filiolium sibiricum*	0±0	1472±1226	0±0	0±0
*Cephalanoplos segetum*	0±0	57±57	0±0	0±0
*Calamagrostis epigejos*	0±0	57±57	0±0	0±0
*Chenopodium acuminatum*	1132±709	170±170	170±98	0±0

Values are mean±s.e.

**Table 2:** ANOVA of the effects of management regimes on each variable for the soil seed bank, aboveground vegetation and soil parameters.

	F	df.N, df.D	P	Mean square
Species richness of SB	1.199	3,8	0.370	0.091
Seed density of SB	12.794	3,8	0.002	1.178
Shannon index of SB	1.297	3,8	0.340	0.3
Shannon index of Vegetation	4.008	3,8	0.052	0.3
SOC	8.974	3,8	0.006	2.1
AN	5.418	3,8	0.025	1847.8
AP	3.226	3,8	0.082	11.6
TK	4.449	3,8	0.093	5.8
Na^+^	23.480	3,8	0.000	23491.4
Cl^-^	47.386	3,8	0.000	10265.4
SO_4_^2-^	1.533	3,8	0.279	774.2
pH	2.258	3,8	0.159	0.2
EC	1.970	3,8	0.197	24043. 8
RSC	4.150	3,8	0.048	52352.2
Alkalinity	5.040	3,8	0.030	53777.8
SAR	34.545	3,8	0.000	464.3
ESP	3.040	3,8	0.093	5.8

Note: df.N represent the numerator degrees of freedom; df.D denominator degrees of freedom. SB represents soil seed bank.

**Fig 2:**
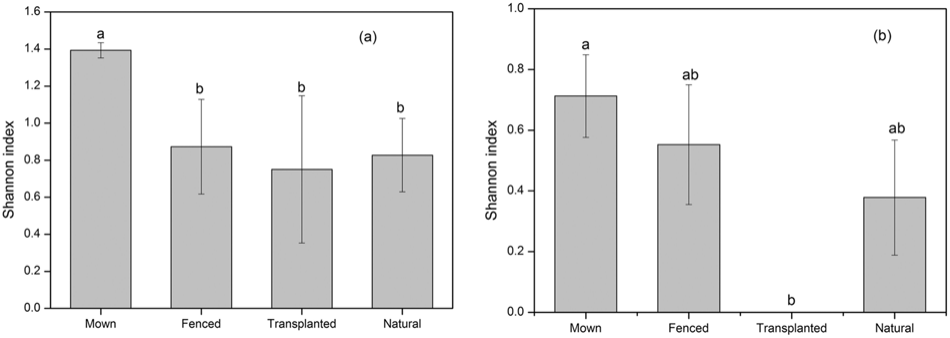
Diversity indices of the soil seed bank (a) and aboveground vegetation (b) in each of the four management regimes of Mown, Fenced, Transplanted and Natural grasslands. Bars with the same lower case letters indicate no significant differences (p > 0.05) between different management regimes based on Tukey’s test. Error bars are mean ± s.e.

Soil seed bank diversity in the Mown grassland was slightly higher relative to the other three management regimes (Fig 2a), although no significant differences in the Shannon diversity index (F_3,8_ = 1.297, p = 0.340, Table 2) was found among the four habitats.

### Vegetation composition

A total of 15 species were recorded from the aboveground vegetation (Table 3), belonging to eight families (Poaceae, Asteraceae, Chenopodiaceae, Cyperaceae, Polygonaceae, Brassicaceae, Fabaceae, and Umbelliferae). The total number of plants/m^2^ did not significantly differ between the four management regimes (F_3,8_ = 0.834, p = 0.512, Table 2). *Leymus chinensis* was the dominant species in all four treatments and there were also no significant differences in density for this species (F_3,8_ = 3.217, p = 0.08, Table 2). However, the lowest density of *L*. *chinensis* was found in mown grassland, which was significantly lower than that in the Fenced and Transplanted habitat.

**Table 3:** The aboveground vegetation density under each of the four management treatments of Mown, Fenced, Transplanted and Natural (control) in the Songnen grassland, Northeastern China, (No./m^2^).

Species	Mown	Fenced	Transplanted	Natural
	No./m^2^ (%)	No./m^2^ (%)	No./m^2^ (%)	No./m^2^ (%)
*Leymus chinensis*	491±223 (44.1±15.8)	1112±141 (19.9±11.1)	1017±143 (100.0±0.0)	735±94 (84.2±8.3)
*Puccinellia chinampoensis*	0±0 (0)	29±7 (2.1±0.7)	0±0 (0)	0±0 (0)
*Chloris virgata*	0±0 (0)	12±12 (0.9±0.9)	0±0 (0)	0±0 (0)
*Setaria viridis*	897±547(51.8±16.8)	185±185 (13.3±13.3)	0±0 (0)	0±0 (0)
*Polygonum sibiricum*	0±0 (0)	13±5 (1.0)	0±0 (0)	0±0 (0)
*Lepidium densiflorum*	30±16 (1.5±1.3)	0±0 (0)	0±0 (0)	0±0 (0)
*Artemisia capillaris*	0±0 (0)	0±0 (0)	0±0 (0)	3±3 (0.4±0.4)
*Phragmite saustralis*	24±16 (1.2±1.0)	3±3 (0.2±0.2)	0±0 (0)	0±0 (0)
*Chenopodium acuminatum*	2±1 (0.1±0.1)	0±0 (0)	0±0 (0)	0±0 (0)
*Sonchus arvensis*	0±0 (0)	0±0 (0)	0±0 (0)	164±109 (6.1±6.1)
*Calamagrostis epigejos*	0±0 (0)	12±12 (0.9±0.9)	0±0 (0)	0±0 (0)
*Lespedeza bicolor*	12±8 (1.0±1.0)	0±0 (0)	0±0 (0)	0±0 (0)
*Taraxacum erythropodium*	0±0 (0)	25±3 (1.8±0.3)	0±0 (0)	0±0 (0)
*Saposhnikovia divaricata*	9±5 (0.3±0.3)	0±0 (0)	0±0 (0)	0±0 (0)
*Scirpus triqueter*	0±0 (0)	0±0 (0)	0±0 (0)	41±41 (9.3±9.3)

Values are mean ± m.s. (n = 3)

The Shannon diversity index for aboveground vegetation differred between the four treatments and this was marginally non-significant (Fig 2, Table 2). The highest Shannon diversity index was observed in the Mown grassland, while the lowest value of zero was recorded in the Transplanted habitat where only *L*. *chinensis* was present.

### Soil characteristics

**Fig 3:**
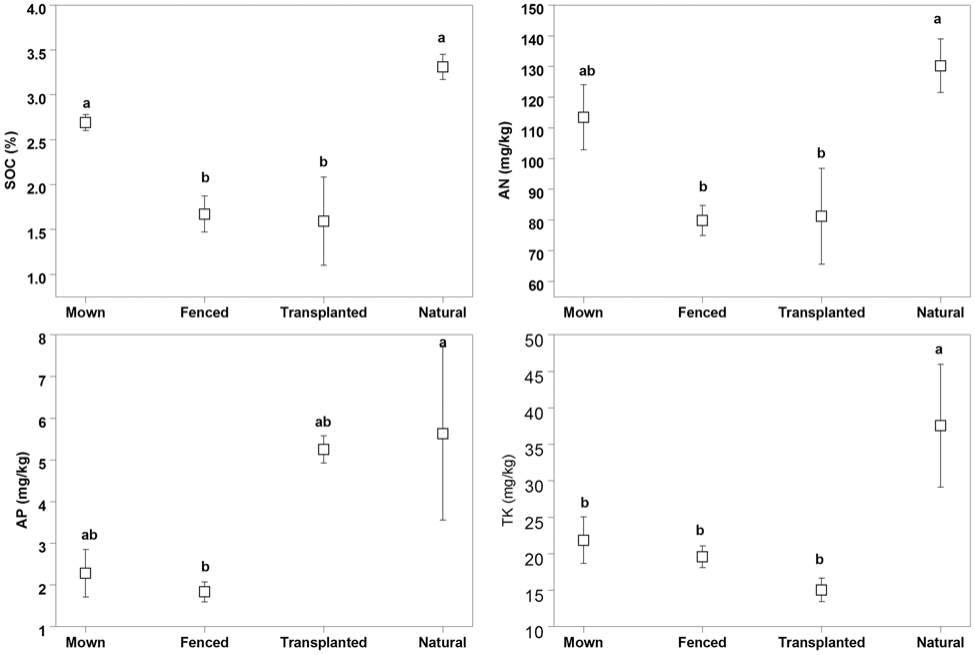
Comparisons of soil nutrients, soil organic carbon (SOC), available nitrogen (AN), available phosphorus (AP) and total potassium (TK) among the four different management regimes of Mown, Fenced, Transplanted and Natural (control). Bars with the same lower case letters indicate no significant differences (p > 0.05) between different management regimes based on Tukey’s test. Error bars are mean ± s.e. (n = 3).

**Fig 4:**
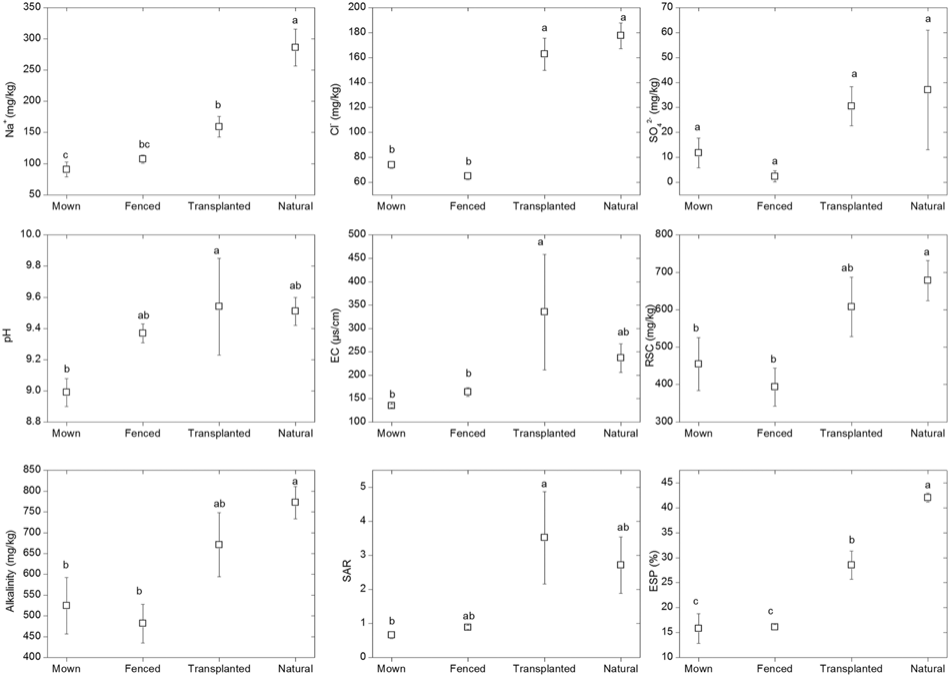
Comparisons of Na^+^, Cl^-^, SO_4_^2-^, soil pH, electrical conductivity (EC), residual sodium carbonate (RSC), alkalinity (CO32-+HCO3-), sodium adsorption ratio (SAR) and exchangeable sodium percentage (EXP) among the four different management regimes of Mown, Fenced, Transplanted and Natural (control). Bars with the same lowercase letters indicate no significant differences (p > 0.05) between different management regimes based on Tukey’s test. Error bars are mean ± s.e. (n = 3).

The ANOVA results for nutrient contents and saline-alkaline parameters in the soil (0–10 cm depth) among the four treatments are shown in Table 2 and Fig 3. The highest content of SOM, AN, AP and TK were all observed in the Natural (Control) treatment which were significantly higher than those in the Fenced treatment. No significant differences were observed in AN, AP and TK among Mown, Fenced or Transplanted grassland sites. The content of Na^+^, Cl^-^, RSC and alkalinity and also ESP in the Natural treatment was significant higher than those in the Mown and Fenced habitats (Fig 4). Natural and Transplanted grasslands had relatively similar soil parameters, such as Na^+^, Cl^-^, SO_4_^2-^, pH, EC, RSC, SAR, alkalinity and ESP.

### Height and seed production of *Leymus chinensis*

The height of *L*. *chinensis* was significantly different among the four different management regimes (F_3,8_ = 22.386, p < 0.001) (Table 4). The greatest height (74.2 cm) was observed in the Transplanted treatment, following by the Natural treatment (53.9 cm), whist heights of just 25.6 and 29.8 cm were observed in the Mown and Fenced treatments. The highest seed set rate of 53.7% was found in the Transplanted site, while only 20.5% and 6.9% respectively were recorded in the Fenced and Natural sites. No spikelets were found in the Mown site.

**Table 4:** Growth and seed setting characteristics of *L. chinensis* under each of the four management treatments of Mown, Fenced, Transplanted and Natural (control) grasslands.

	Mown	Fenced	Transplanted	Natural
Plant height (cm)	29.8±5.0c	25.6±1.5c	74.2±7.4a	53.9±3.0b
Seed setting rate(%)	/	20.5±3.2b	53.7±3.6a	6.9±1.7c
No. seeds/spikelet	/	46.2±2.9b	51.1±4.6b	87.7±5.8a
Length of spikelet (cm)	/	8.4±0.4b	12.7±0.5a	11.4±0.6ab

/represents no data, as no *L*. *chinensis* seeds were produced at the mown sites. Values are mean ± s.e. (n = 3). Different lowercase letters in the same row represent significant differences at 0.05 level between management regimes based on Tukey’s test.

### Similarity between the soil seed bank and above-ground vegetation

**Fig 5:**
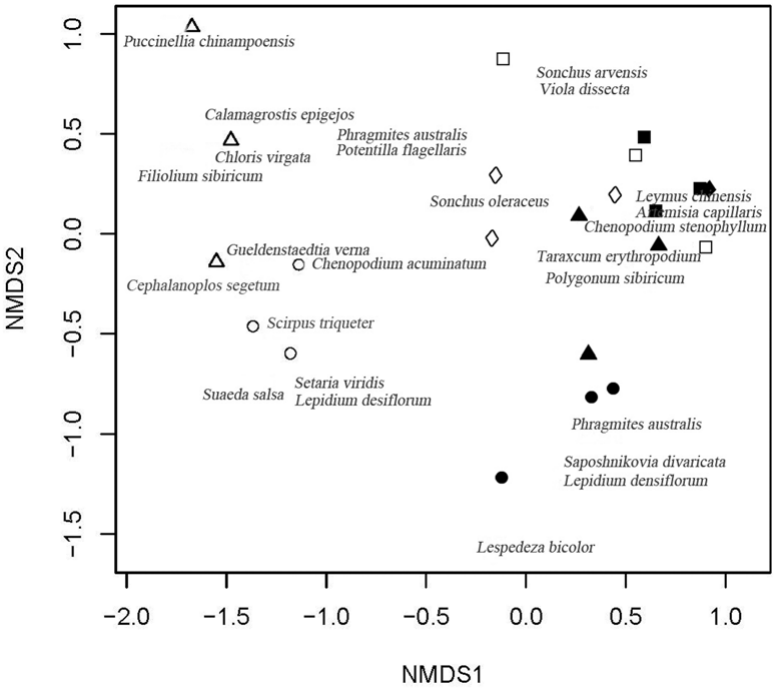
Two-dimensional nonmetric multidimensional scaling (NMDS) ordination of soil seed banks and above-ground vegetation in *L*. *chinensis* communities with different management regimes in the Songnen Plain, northeastern China (stress value = 0.12). Ordination is based on species abundance data. In the graph, the dark circles ●represent vegetation in the Mown treatment; the dark triangle ▲ represents vegetation in the Fenced treatment;the dark diamand ◆ represents vegetation in the Transplanted treatment; the dark square ■ represents vegetation in the Natural treatment;the open circle ○ represents the soil seed bank in the Mown treatment; the open triangle △ represents the soil seed bank in Fenced treatment; the open diamond ◇ represents the soil seed bank in the Transplanted treatment; and the open square □ represents the soil seed bank in the Natural (control) treatment. Note: Three sites of the Transplanted grassland vegetation were overlapped in one in the Figure which was the same as one site of the Natural vegetation site.

In the ordination graph (Fig 5), the first axis of the NMDS clearly separated the soil seed bank groups of the Fenced and Mown treatments from the other two treatments. The above-ground vegetation was grouped closely for all treatments, however, the Mown treatment was separated by a small distance along axis 2. Soil seed banks of the Natural and Transplanted treatments were similar to all of the above-ground vegetation groups except for that of the Mown treatment. Some species were common in the soil seed banks of particular treatment types, such as *Puccinellia chinamponensis*, *Chloris virgata*, *Filiolium sibircum* (Fenced) and *Scirpus triqueter* (Mown).

### Relationship between soil characteristics and soil seed bank and vegetation composition

In the NMDS analysis, the seed density of species in the soil seed banks was significantly related to the soil variables (vectors) including Na^+^, Cl^-^, RSC,alkalinity, ESP and AP with p values of 0.002, 0.002, 0.027, 0.025, 0.001, and 0.005 respectively. However, some soil variables such as SO_4_^2-^, pH, EC, SAR, SOC and AN were not significantly related to seed density. Similarly, variables of Na^+^, Cl^-^, RSC, alkalinity, ESP, AP significant were also related to aboveground vegetation distribution, with p values of 0.032, 0.002, 0.040, 0.035, 0.008, and 0.038 respectively while SO_4_^2-^, pH, EC, SAR, SOC and AN were not.

**Fig 6:**
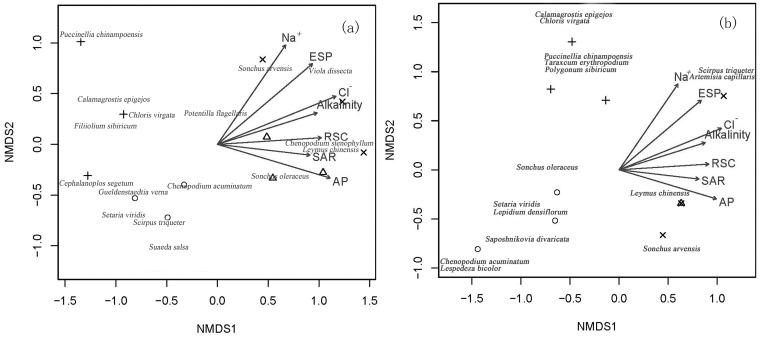
Relationship between soil characteristics and (a) seed densities of species germinating from soil seed banks and (b) abundance of aboveground vegetation in each of the management regimes including Mown, Fenced, Transplanted and Natural (control). Variation in species density, both in the soil seed banks and the aboveground vegetation, were related to Na^+^,Cl^-^,RSC,alkalinity, ESP and AP at p < 0.05 level. ○ represents the Mown treatment, + represents the Fenced treatment, Δ represents Transplanted treatment, and × represents the Natural (control) treatment.

The soil properties related with seed densities of species in the soil seed bank and vegetation structure were shown in the NMDS ordination graphs (Fig 6). The concentrations of Na^+^, ESP, Cl^-^, and alkalinity increased in the upper quadrant of the graph and was related to the density of *Sonchus arvensis* and *Viola dissecta* in the soil seed bank, and to the density of *Scirpus triqueter* and *Artemisia capillaris* in the vegetation. The RSC, SAR, and AP concentrations increased along with *L*. *chinensis* in both soil seed bank and aboveground vegetation.

## Discussion

Our results demonstrated that management regimes significantly affected the soil seed density in saline-alkaline grasslands. The aboveground vegetation community structures and some aspects of the soil physical-chemical properties were also different between the grasslands under the four management regimes. We confirmed that the variation in soil seed bank density and diversity between treatments were strongly related with the aboveground vegetation composition and soil characteristics in agreement with previous studies [26,30]. Although *L*. *chinensis* was the most abundant species represented in the aboveground vegetation in all management treatments, it only occurred in the soil seed bank in the Transplanted and Natural treatment sites. Judging from the plant growth, seed production and the presence of buried seeds of this target species, the Transplanted treatment could be the best method for the restoration of *L*. *chinensis* dominated grasslands.

We found high variability in seed bank density ranging from 2265 seeds/m^2^ (transplanted) to 30348 seeds/m^2^ (Fenced) which was well within the range of grassland ecosystems (10^3^–10^6^ seeds/m^2^) noted in other studies [4,17]. However, so far, few studies have been carried out in the saline-alkaline grasslands. Valkó et al. [30] detected 30,104–51410 seeds/m^2^ in the alkaline grassland in Hungary and Ma et al. [27] found 1558 seeds/m^2^ in a saline alkaline meadow in the eastern Tibetan Plateau, China. The high variability found in our study is very likely to have been affected by the long-term implementation of the four different management treatments. However, we cannot discount that seed production and reproductive mechanisms of some of the other common species, as well as the level of disturbance and deterioration experienced within each site, may also contribute to these differences.

The influence of restoration management regimes on soil seed bank composition have been assessed in several previous studies [1,10,12], although results have varied markedly. Human-implemented management regimes are often considered to be effective methods for restoration and can promote the maintenance of high plant species diversity [4]. In this study, the highest species richness in the soil seed bank was obtained at the Mown site, although it showed no significant differences with the other treatments (Fig 1a). Compared with the Natural treatment, seed bank densities from Mown and Fenced treatment were significantly higher while that of the Transplanted habitat was significantly lower (Fig 1b). Higher seed density in the Fenced sites was mainly caused by the presence of *Puccinellia chinampoensis* which showed huge seed production. Consistent with our results, Ma et al. [44] also demonstrated significantly higher seed density and species richness in a fenced treatment compared to the control site in an alpine meadow. However, Shang et al. [10] reported that species richness in the soil bank declined by 60% of the original species richness in fenced treatments, while mown sites produced a decline of 20% in calcareous grasslands. Therefore, with no uniform conclusion for the effect of different management regimes on overall species richness, because of the likely differences grassland types and disturbance characteristics, we suggest that treatments for restoration should be selected based on the response of the group of species, or dominant species, that are the target of restoration efforts.

In the Transplanted treatment, transplanting methods created suitable microsites, which in turn accelerated the seedling establishment and survival of *L*. *chinensis*, which lead to high aboveground density and taller plants (Tables 2 and 3). However, the competition for light between plants might likely induce the decline of species richness that was recorded, as has been found after abandonment in other grassland studies [45]. In our Transplanted treatment, the height of the dominant species *L*. *chinensis* increased by 38% compared to the Natural treatment, a large difference which is likely to have allowed far less light penetration to the soil surface. However, the height of *L*. *chinensis* in Mown and Fenced treatments decreased by 44.7% and 52.5%, respectively in comparison to the Natural treatment. Jacquemyn et al. [46] also showed the similar results in calcareous grasslands. The density of the dominant *L*. *chinensis* may partly explain the differences of total species abundances observed between the Transplanted and Fenced or Mown habitats.

Seed production of the aboveground vegetation, an important source for the soil seed bank, was strongly related to the variation in soil seed bank characteristics [47,48], and it is likely that implemented management regimes directly affect species composition of the vegetation more dramatically than the seed bank [49]. In this study, the reproduction of the target species *L*. *chinensis* differed greatly under different management regimes. The sexual reproduction of *L*. *chinensis* was clearly inhibited by the mowing behavior and few spikelets were found in the Mown habitats. Seed production was reduced after mowing due to the lower numbers of tillers and inflorescences per plant, spikelets per inflorescence, and seed quantity per spikelet [47]. In contrast, the Transplant treatment promoted both the vegetative and reproductive growth of *L*. *chinensis*.

The absence of *L*. *chinensis* seed in the soil seed bank within the Fenced treatment was an unexpected finding, because seed production was high compared to the Natural treatment (Table 4). This might be due to the limitation of our sampled area which, if seed densities were low, could have failed to detect the species within the soil. On a more general note regarding species diversity within the seed bank, our estimate of seed density is only a snapshot, taken at one time during the year [50]. Sampling time has proven to be a particularly important factor influencing species composition in the soil seed bank. We acknowledge that one-off samples of the seed bank, above-ground vegetation and, particularly, edaphic conditions may therefore have produced some limitations within our results. For example, it is unlikely that our soil samples included all species of seeds because, within any community, there are species with different seed-producing phenologies. It is also possible that the dormancy or germination requirements of some species may not have been met during the treatment period. Multiple sampling times throughout the year would permit the tracking of seeds of various species in the seed bank [7], however, was beyond the scope of this study. In the future, to better address the question of restoration over time, all sampling should occur at intervals throughout the year.

In this study, we showed that Na^+^, Cl^-^, RSC, alkalinity, ESP, and AP were significantly related with soil seed bank composition (Fig 6a) while pH, EC, SAR, SOC and AN displayed no relationship. Hegazy et al. [26] also demonstrated significant associations between the composition of the seed bank and the edaphic factors such as CaCO_3_, electrical conductivity, organic carbon and soil texture. However, Valkó et al. [30] showed no significant effect of salinity (EC) on the soil seed bank composition. In accordance with Janssens et al. [51], our results also showed that phosphorus content is compatible with plant diversity; while some other factors (i.e. pH, organic matter, total nitrogen and calcium) do not show a significant relationship with plant diversity.

Acting as a propagule reservoir, utilization of the soil seed bank has proved to be a potentially important method for the ecological restoration of many degraded ecosystems. For example, soil seed banks have been reported to contribute to more than 40% of emerging seedlings in acid [52] and calcareous grasslands [50]. However, some researchers have found that the soil seed bank plays only a minor role, or has no effect on vegetation restoration [53,54]. In our study, although the Fenced and Mown treatments displayed a relatively high seed density and diversity within the soil seed bank, the absence of the target species *L*. *chinensis*, indicates that affective restoration from the seed bank by these methods would be limited, at least in the short-term, especially because a grassland dominated by *L*. *chinensis* is the desired management outcome. This does not discount the fact that seed banks may play a major role in the future, as has been shown in some studies [1,21], but only after they have re-established over several decades and vegetation is subsequently well-developed. As well as the availability of seeds, successful re-establishment from the seed bank is also dependent upon a combination of many other factors, such as the availability of favorable microsites suitable for germination and establishment of seedlings [13], and appropriate temperature and rainfall conditions for overcoming dormancy [54].

While the key goal of restoration of grassland (and other) ecosystems is usually to re-establish vegetation, management strategies should also take into account the possibility of restoring soil seed bank processes and dynamics [53]. In this study, the Transplanted treatment resulted in successful vegetation restoration, especially in severely degraded grasslands, and also increased the seed density of *L*. *chinensis* in the soil seed bank. What was particularly interesting is that compared with the Transplanted treatment, *L*. *chinensis* plants in the Natural (control) treatment displayed a tendency for lower seed set rates and smaller plants. Matus et al. [1], as well as numerous other grassland studies, found that the density of viable seeds decrease with increasing successional age, suggesting that the older Natural site had experienced a reduction in seed input. Evidence in our study showed that, after 10 years, both Mown and Fenced treatment had higher species richness, but that the climax species of *L*. *chinensis* was much smaller in size and abundance compared to the other two treatments. This suggests that management approaches, such as fencing, might only see restoration of climax vegetation after a few decades. Thus, for a faster and more efficient return to *L*. *chinensis* dominated systems, transplanting is a preferred method for restoration.

To date, transplanting adult plants or seedlings have been shown to be successful in restoration of coastal communities [55–57], wetland meadows [58, 59] and grassland ecosystems [60, 61]. For example, De Steven and Sharitz [57] attained 15–85% cover two years after transplanting, and Aradottir [60] demonstrated that transplanting of native turfs was a promising method for establishing a diverse range of native species on disturbed areas. As plants mature from seed to rhizome-bearing adults, once established, plants become more tolerant to a broad range of seasonal drying and flooding conditions [59], and salinity [62]. The feasibility of a transplant approach is obviously dependent on the area requiring restoration and other options such as fencing may still be viable for much larger areas. However, the development of soil seed banks in the longer-term under this management option requires further study before stronger recommendations can be made.
